# Amino-Terminal β-Amyloid Antibody Blocks β-Amyloid-Mediated Inhibition of the High-Affinity Choline Transporter CHT

**DOI:** 10.3389/fnmol.2017.00361

**Published:** 2017-11-07

**Authors:** Leah K. Cuddy, Claudia Seah, Stephen H. Pasternak, R. Jane Rylett

**Affiliations:** ^1^Molecular Medicine Research Laboratories, Robarts Research Institute, University of Western Ontario, London, ON, Canada; ^2^Department of Physiology and Pharmacology, Schulich School of Medicine & Dentistry, University of Western Ontario, London, ON, Canada; ^3^Department of Neurology, Feinberg School of Medicine, Northwestern University, Chicago, IL, United States; ^4^Department of Clinical Neurological Sciences, Schulich School of Medicine & Dentistry, University of Western Ontario, London, ON, Canada

**Keywords:** protein trafficking, cholinergic, β-amyloid, Alzheimer’s disease

## Abstract

Alzheimer’s disease (AD) is a common age-related neurodegenerative disorder that is characterized by progressive cognitive decline. The deficits in cognition and attentional processing that are observed clinically in AD are linked to impaired function of cholinergic neurons that release the neurotransmitter acetylcholine (ACh). The high-affinity choline transporter (CHT) is present at the presynaptic cholinergic nerve terminal and is responsible for the reuptake of choline produced by hydrolysis of ACh following its release. Disruption of CHT function leads to decreased choline uptake and ACh synthesis, leading to impaired cholinergic neurotransmission. We report here that cell-derived β-amyloid peptides (Aβ) decrease choline uptake activity and cell surface CHT protein levels in SH-SY5Y neural cells. Moreover, we make the novel observation that the amount of CHT protein localizing to early endosomes and lysosomes is decreased significantly in cells that have been treated with cell culture medium that contains Aβ peptides released from neural cells. The Aβ-mediated loss of CHT proteins from lysosomes is prevented by blocking lysosomal degradation of CHT with the lysosome inhibitor bafilomycin A1 (BafA_1_). BafA_1_ also attenuated the Aβ-mediated decrease in CHT cell surface expression. Interestingly, however, lysosome inhibition did not block the effect of Aβ on CHT activity. Importantly, neutralizing Aβ using an anti-Aβ antibody directed at the N-terminal amino acids 1–16 of Aβ, but not by an antibody directed at the mid-region amino acids 22–35 of Aβ, attenuates the effect of Aβ on CHT activity and trafficking. This indicates that a specific N-terminal Aβ epitope, or specific conformation of soluble Aβ, may impair CHT activity. Therefore, Aβ immunotherapy may be a more effective therapeutic strategy for slowing the progression of cognitive decline in AD than therapies designed to promote CHT cell surface levels.

## Introduction

Alzheimer’s disease (AD) is a neurodegenerative disorder defined by progressive and irreversible cognitive decline. Cognitive and attentional processes impaired in AD are mediated by cholinergic neurons with release of the neurotransmitter acetylcholine (ACh; Sarter and Parikh, 2005). After binding to receptors, ACh is hydrolyzed by acetylcholinesterase to choline and acetate with choline then taken up into cholinergic presynaptic terminals by the high-affinity choline transporter (CHT) to serve as substrate for ACh synthesis (Haga and Noda, 1973; Kuhar and Murrin, 1978; Rylett and Schmidt, 1993). Recycling of CHT proteins between the cell surface and endosomal compartments maintains plasma membrane CHT levels, thereby regulating choline uptake activity (Black and Rylett, 2012). CHT proteins internalize constitutively from the plasma membrane by a clathrin-dependent mechanism and either recycle back to the cell surface or move through late endosomes to lysosomes for degradation (Cuddy et al., 2012).

Two major features of AD pathology are dysfunction of cholinergic transmission early in the course of the disease and the progressive accumulation of β-amyloid (Aβ) peptides produced by cleavage of amyloid precursor protein (APP), with a link having been established between these two aspects of AD pathology. Cholinergic transmission promotes the non-amyloidogenic α-cleavage of APP through ACh stimulation of muscarinic receptors (Nitsch et al., 1992), while APP plays a role in cholinergic neurons by regulating the presynaptic localization of CHT proteins and their internalization from the cell surface (Wang et al., 2007). Importantly, it has been shown in experiments using synthetic preparations of Aβ peptides that oligomeric Aβ can negatively regulate ACh synthesis and release by inhibiting high-affinity choline uptake (Pedersen et al., 1996; Auld et al., 1998; Kar et al., 1998; Parikh et al., 2014); one study reported enhanced choline uptake activity in synaptosomes and neural cells exposed acutely to oligomeric Aβ_1–42_, but links this to reduced ACh release (Bales et al., 2006). Following depolarization of synaptosomes, incubation with Aβ_1–40_ decreases binding of the CHT ligand [^3^H]hemicholinium-3 ([^3^H]HC-3), giving an indirect measurement of transporter reduction at the cell surface (Kristofiková et al., 2001). Thus, accumulation of Aβ peptides in brain may inhibit CHT activity, decreasing ACh synthesis and impairing cholinergic transmission.

Evidence suggests that soluble Aβ peptides promote the disease process by several mechanisms, including disrupting synaptic transmission and impairing long-term potentiation (Walsh et al., 2002; Townsend et al., 2006; Chen et al., 2013). Accordingly, passive immunization approaches using Aβ antibodies to neutralize and facilitate clearance of soluble Aβ peptides are being evaluated clinically for the treatment of AD (Schenk, 2002; Panza et al., 2011). Antibodies targeting different epitopes in the N-terminus, mid-region and C-terminus of Aβ peptides have been designed, with most preclinical studies focusing on N-terminal Aβ antibodies such as Bapineuzumab (Miles et al., 2013) and mid-region Aβ antibodies such as Solanezumab (m266; DeMattos et al., 2001). Some Aβ antibodies can bind to and effectively reduce either soluble and fibrillar forms of Aβ, thereby preventing synaptic degeneration and cognitive deficits in animal models of AD (Bard et al., 2000; Schroeter et al., 2008; Pul et al., 2011; Zago et al., 2012).

The purpose of the present study was to investigate the effect of Aβ peptides released into conditioned medium (CM) from neural cells expressing Swedish mutant APP (CM-APP_Swe_) on CHT trafficking and activity, and to determine whether this is altered by anti-Aβ antibodies. We found recently that expression of APP_Swe_ in neural cells decreases CHT function when compared to wild-type APP with this related to increased APP_Swe_ processing (Cuddy et al., 2015). We now make the novel observation that treatment of neural cells with CM that contains Aβ peptides from cells expressing APP_Swe_ decreases CHT co-localization with the early endosome marker EEA1 and lysosome marker LAMP-1, suggesting that Aβ-mediated inhibition of CHT function is related to a loss of CHT proteins from endocytic recycling compartments. In support of this, we found that the lysosome inhibitor bafilomycin A1 (BafA_1_) attenuates Aβ-mediated inhibition of CHT cell surface expression. Interestingly, however, lysosome inhibition did not block the effect of Aβ on CHT activity. Importantly, inhibition of CHT function by Aβ peptides was blocked by an antibody directed at the N-terminal amino acids 1–16 of Aβ (anti-Aβ[1–16]), but not by an antibody directed at the mid-region amino acids 22–35 of Aβ (anti-Aβ[22–35]).

## Materials and Methods

### Materials

Rabbit anti-β-amyloid (22–35) antibody (anti-Aβ[22–35]) and protease inhibitor cocktail were from Sigma-Aldrich (St. Louis, MO, USA). Anti-β-amyloid 1–16 (6E10) mouse monoclonal antibody (anti-Aβ[1–16]) was from Covance (Princeton, NJ, USA). Rabbit polyclonal anti-actin antibody was from Santa Cruz Biotechnology (Santa Cruz, CA, USA). [Methyl-^3^H]Choline chloride (88.5 Ci/mmol) was from PerkinElmer Life Sciences (Boston, MA, USA). SH-SY5Y human neuroblastoma cells were from American Type Culture Collection (Manassus, VA, USA), and Invitrogen (Burlington, ON, Canada) supplied fetal bovine serum (FBS), Lipofectamine 2000, OptiMEM, culture media and reagents, AlexaFluor-488 donkey anti-mouse IgG and AlexaFluor-647 goat anti-rabbit IgG antibodies. Enhanced ChemiLuminescence (ECL) immunoblot reagent, Protein G Sepharose and Biodegradable Scintillant were from GE Healthcare Life Sciences (Baie d’Urfé, QC, Canada). Clarity ECL was from BioRad (Mississauga, ON, Canada). Polyclonal CHT antibody was raised in rabbits to the antigenic peptide DVDSSPEGSGTEDNLQ conserved at the C-terminus of human and rat CHT (Genemed Synthesis, San Antonio, TX, USA); this peptide was conjugated to KLH carrier protein by an N-terminal cysteine residue (Pinthong et al., 2008). CHT-specific IgG was affinity-purified in our laboratory from crude antiserum on NHS-Sepharose (GE Healthcare) to which antigenic peptide had been coupled as the binding element. Peroxidase-conjugated goat anti-rabbit IgG and peroxidase-conjugated goat anti-mouse IgG were from Jackson ImmunoResearch Laboratories (West Grove, PA, USA).

### Cell Transfection and Selection of Cell Lines

Full-length rat CHT cDNA ligated to pSPORT was a gift from Dr. T. Okuda (Okuda and Haga, 2000); a FLAG-epitope tag (DYKDDDDK) was added to the amino-terminus by PCR and the resulting cDNA ligated to pcDNA3.1. SH-SY5Y cells were transfected with FLAG-CHT plasmid by Lipofectamine 2000. Stable transformants (SY5Y-CHT cells) were selected using 500 μg/ml geneticin (G418) for 4 weeks, and then grown in complete medium (DMEM containing 10% FBS, 100 U/ml penicillin, and 100 μg/ml each of streptomycin and G418). SH-SY5Y cell differentiation was induced by addition of 10 μM RA (all-trans-retinoic acid) for 3 days, during which time cells underwent morphological and biochemical differentiation. For transient transfection, cells were treated with RA for 3 days, then immediately before transfection culture medium was changed to complete medium without antibiotics. At the time of transfection, a ratio of 1 μg plasmid DNA in 100 μl OptiMEM was added to 100 μl OptiMEM containing 2.5 μl Lipofectamine 2000, then incubated for 20 min at room temperature. This mixture was added to cell monolayers in antibiotic-free medium and incubated for 4–6 h. At the end of this incubation, culture medium was replaced with complete medium containing RA and grown for an additional 24 h.

### Preparation of Conditioned Medium (CM)

SY5Y-CHT cells were grown to near confluency on 100 mm dishes in complete medium with 10 μM RA for 3 days. Cells were transiently transfected with 9 μg per dish of either APP_Swe_ plasmid DNA or the empty vector pcDNA3.1 using Lipofectamine 2000. The full-length human isoform 695 APP_Swe_ plasmid, generated by Dr. D. Selkoe (Young-Pearse et al., 2007), was obtained from Addgene (plasmid 30145). Following transfection, culture medium was replaced with 5 mL complete medium containing 10 μM RA per 100 mm dish and grown for an additional 24 h to condition the medium. This CM collected from vector-expressing cells (CM-vector) and APP_Swe_-expressing cells (CM-APP_Swe_) was cleared of cells and cellular debris by centrifugation at 300× *g* at 4°C for 10 min and either used immediately or stored at −80°C. Storage at −80°C does not alter the Aβ concentration in CM based on measurements using a human Aβ_1–42_ ELISA or by Aβ immunoblot profile. Two separate batches each of CM-vector and CM-APP_Swe_ were collected from successive passages of cells (250 mL total per collection from 50 culture plates) for use in these studies. The consistency in Aβ concentration and Aβ immunoblot profile was confirmed between CM batches using Aβ_1–42_ ELISA to measure Aβ_1–42_ concentration and Aβ immunoprecipitation from CM to assess the amount and apparent molecular masses of the Aβ peptides recovered.

### Immunoprecipitation and Neutralization of Conditioned Medium

In some experiments, Aβ peptides were immunoprecipitated from CM-vector and CM-APP_Swe_. CM was first pre-cleared with 15 μL/mL of washed Protein G Sepharose for 1 h at 4°C, then Protein-G Sepharose and non-specifically bound proteins were removed from CM by centrifugation at 2500× *g* for 5 min. Cleared CM supernatant was incubated with 5 μg/mL of either negative control anti-HA antibody, anti-Aβ[1–16] or anti-Aβ[22–35] for 1 h at 4°C. Washed Protein-G Sepharose (15 μL/mL) was then added to samples and mixed by rotation for 24 h at 4°C. Protein-G Sepharose with bound proteins were collected by centrifugation and washed three times with lysis buffer to remove non-specifically bound proteins. Proteins were eluted by incubation for 10 min at 55°C with Aβ Laemmli sample buffer (2% SDS, 40% glycerol, 200 mM Tris-HCl, pH 6.8, 0.04% bromophenol blue and 2% β-mercaptoethanol), then separated on 12% SDS-PAGE gels and transferred to polyvinylidene difluoride (PVDF) membranes. Membranes were blocked in 8% non-fat dry milk in wash buffer (phosphate-buffered saline (PBS) with 0.15% Triton X-100) for 1 h, then incubated overnight at 4°C with anti-Aβ[1–16] antibody (1:1000). After washing, membranes were incubated for 1 h in wash buffer containing 8% milk and peroxidase-conjugated goat anti-mouse IgG secondary antibody. Immunoreactive proteins on membranes were detected by chemiluminescence using a Chemidoc Imaging System (BioRad). Membranes were stripped for 20 min at 55°C followed by 5 min at room temperature in stripping buffer (62.5 mM Tris-HCl, pH 6.7, 2% SDS, 0.78% 2-mercaptoethanol), and then washed five times for 30 min in wash buffer before being re-probed with anti-Aβ[22–35] antibody (1:1000).

In experiments where Aβ peptides were neutralized in CM-vector and CM-APP_Swe_, CM was incubated with 5 μg/mL of either negative control anti-HA antibody, anti-Aβ[1–16] antibody or anti-Aβ[22–35] antibody for 24 h at 4°C. This medium was then used to treat SY5Y-CHT cells that had been grown in complete medium containing 10 μM RA for 3 days for a period of 24 h at 37°C.

### Aβ_1–42_ ELISA

The amount of human Aβ_1–42_ released by cells was measured in CM-vector and CM-APP_Swe_ at 24 h following transfection using the human Aβ_1–42_ ELISA kit (Invitrogen), according to the manufacturer’s protocols. In some experiments, CM was incubated for an additional 24 h at 4°C with either anti-HA, anti-Aβ[1–16] or anti-Aβ[22–35] antibody, then Aβ_1–42_ content was measured.

### [^3^H]Choline Uptake Assay

Choline uptake activity was evaluated in SY5Y-CHT cells grown for 24 h in either CM-vector or CM-APP_Swe_ that had been pre-incubated for 24 h with either anti-HA, anti-Aβ[1–16] or anti-Aβ[22–35] antibody. Monolayers of cells were rinsed with warm Krebs-Ringer-HEPES (KRH) buffer (mM: NaCl, 124; KCl, 5; MgSO_4_, 1.3; CaCl_2_, 1.5; glucose, 10; HEPES-NaOH, 20; pH 7.4), then incubated in KRH at 37°C for 15 min. This buffer was aspirated from cells and choline uptake initiated by the addition of KRH buffer containing 0.5 μM [^3^H]choline (0.5 Ci/mmol) either with or without 1 μM HC-3. Uptake was stopped after 5 min by placing cells on ice and washing with ice-cold KRH. Cells were solubilized in 0.1 M NaOH, then aliquots taken for tritium quantification by liquid scintillation counting and protein measurement.

### Cell Surface Protein Biotinylation Assay

SY5Y-CHT cells plated on 100 mm dishes were grown for 24 h in either CM-vector and CM-APP_Swe_ that was immuno-depleted by 24 h treatment with either anti-HA, anti-Aβ[1–16] or anti-Aβ[22–35] antibody. Cells were then washed twice with cold HBSS and placed on ice under cold HBSS to stop protein trafficking. Plasma membrane proteins were biotinylated on ice by incubating with 1 mg/ml sulfo-NHS-SS-biotin in HBSS for 1 h. Unbound biotin was quenched by washing and incubating cells in cold 100 mM glycine in HBSS. After two further washes with HBSS, cells were lysed on ice for 45 min in 1% Triton X-100 lysis buffer. Neutravidin beads were incubated with cell lysates for 1 h at 4°C with gentle mixing to bind biotin-labeled proteins to allow their separation from the non-biotinylated proteins. Beads were then washed with lysis buffer three times and bound proteins were eluted by incubation for 10 min at 55°C with Laemmli sample buffer. Aliquots of biotinylated proteins and total cell lysates were separated on 10% SDS-PAGE gels and transferred to PVDF membranes. Membranes were blocked in 8% non-fat dry milk in wash buffer for 1 h, and then incubated overnight with either anti-CHT (1:3000), anti-actin (1:3000), or anti-calnexin (1:1000) antibody in wash buffer with 8% non-fat milk. After washing, membranes were incubated for 1 h with either peroxidase-conjugated goat anti-rabbit IgG (1:10,000) or peroxidase-conjugated goat anti-mouse IgG (1:10,000) secondary antibody in wash buffer containing 8% milk, and washed again. Immunoreactive proteins were detected by chemiluminescence using a Chemidoc Imaging System (BioRad). Immunopositive bands were quantified by densitometry.

### Confocal Imaging

Digital images of fixed cells were acquired with a Zeiss LSM510-Meta laser-scanning confocal microscope using a 63× magnification oil-immersion objective and magnified three times. Untransfected SY5Y-CHT cells plated on 35 mm dishes were grown for 24 h in CM collected from either vector-expressing or APP_Swe_-expressing SY5Y-CHT cells. Immediately prior to cell treatment, CM was incubated for 24 h with either anti-HA, anti-Aβ[1–16] or anti-Aβ[22–35] antibody. Cells were then washed twice in warm HBSS and formaldehyde-fixed. Fixed cells were incubated first with anti-CHT (1:1000) or anti-LAMP1 (1:200) primary antibodies, then incubated with secondary antibodies rabbit AlexaFluor 647 (1:1000), to label CHT, and mouse AlexaFluor 488 (1:1000), to label LAMP1. Images were then acquired using 488 nm excitation and 505–530 nm emission wavelengths for LAMP1 and 647 nm excitation and 650 nm emission using a long pass filter for CHT. Co-localization analysis was performed on confocal images using Imaris software version 7.7.0 with the Imaris Co-localization module (Bitplane) to examine the co-localization of the brightest 2% of pixels in each channel, as described previously (Lorenzen et al., 2010; Cuddy et al., 2012).

### Data Analysis

Data are presented as the mean ± SEM with *n* values representing the number of independent experiments performed on separate populations of cells; each *n* value was obtained from the average of multiple sample replicates in each experiment. Replicate experiments were performed on cells cultured in successive passages as much as possible to minimize inter-experiment variability, and intra-experiment variability between replicate samples was minimal. GraphPad Prism 5 was used for data analysis. Statistical significance was determined by paired Student’s *t*-test, or between groups using repeated measures one-way analysis of variance (ANOVA) with Tukey’s *post hoc* multiple comparison test or by two-way ANOVA, as appropriate.

## Results

### Soluble Aβ Peptides Are Present in Medium Conditioned by SY5Y-CHT Cells Expressing APP_Swe_

**Figure 1:**
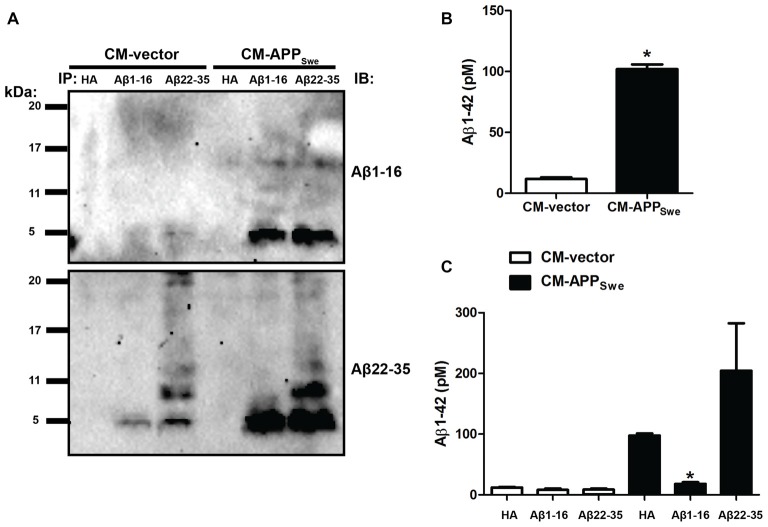
β-amyloid (Aβ) proteins are present in conditioned medium (CM) derived from SY5Y-CHT cells expressing APP_Swe_. **(A)** CM was collected from SY5Y-CHT cells that had been transiently transfected with either vector or APP_Swe_ plasmids and allowed to express for 24 h (CM-vector and CM-APP_Swe_, respectively). Aβ was immunoprecipitated from CM-vector and CM-APP_Swe_ using Aβ antibody directed at amino acids 1–16 of Aβ (anti-Aβ[1–16]) or Aβ antibody directed at amino acids 22–35 of Aβ (anti-Aβ[22–35]) and Protein G Sepharose. Proteins were solubilized, separated by SDS-PAGE and transferred to polyvinylidene difluoride (PVDF) membranes. Membranes were probed with anti-Aβ[1–16] (top panel) and anti-Aβ[22–35] (lower panel). Representative immunoblots show Aβ proteins present in CM-vector and CM-APP_Swe_. CM-vector and CM-APP_Swe_ were incubated with anti-HA antibody and Protein G Sepharose as a negative control (lanes 1 and 4). The immunoblots shown are representative of data obtained from three independent experiments. **(B)** Human Aβ_1–42_ concentration in CM-vector and CM-APP_Swe_ as measured by ELISA. Data are the mean ± SEM of five independent experiments, with statistical analyses performed using a student’s paired *t*-test (*p ≤ 0.05). **(C)** CM-vector and CM-APP_Swe_ were incubated with either HA, anti-Aβ[1–16] or anti-Aβ[22–35] for 24 h and Aβ_1–42_ concentration was measured by ELISA. Incubating CM-APP_Swe_ with anti-Aβ[1–16] significantly lowers Aβ_1–42_ concentration compared to that found in CM-APP_Swe_ incubated with either anti-HA or anti-Aβ[22–35] antibody. Data are the mean ± SEM of five independent experiments, with statistical analyses performed using a repeated-measures one-way analysis of variance (ANOVA) with Tukey’s *post hoc* multiple comparisons test (*p ≤ 0.05).

The purpose of the present study was to investigate the effect of endogenously produced Aβ peptides on CHT function. To this end, we measured CHT activity and trafficking in untransfected SY5Y-CHT cells treated with Conditioned Media (CM) derived from SY5Y-CHT cells that transiently expressed either empty vector or APP_Swe_ (CM-vector and CM-APP_Swe_, respectively). Therefore, we determined the amount and apparent molecular masses of soluble Aβ peptides present in CM-vector and CM-APP_Swe_. Aβ peptides were recovered from CM-vector and CM-APP_Swe_ by immunoprecipitation using either anti-HA antibody as a negative control, anti-Aβ antibody directed at amino acids 1–16 at the N-terminus of Aβ (anti-Aβ[1–16] antibody) or anti-Aβ antibody directed at amino acids 22–35 in the mid-region of Aβ (anti-Aβ[22–35] antibody). Figure 1A shows a representative immunoblot that was probed for Aβ peptides using anti-Aβ[1–16] antibody (top panel), then stripped and re-probed for Aβ peptides using anti-Aβ[22–35] antibody (bottom panel). No Aβ peptides were immunoprecipitated from CM-vector or CM-APP_Swe_ with the anti-HA antibody, whereas strong Aβ immunoreactive bands with masses of approximately 4-kDa were recovered from CM-APP_Swe_ immunoprecipitated with either anti-Aβ[1–16] or anti-Aβ[22–35] antibody (Figure 1A). Faint immunoreactive Aβ bands with masses of about 4-kDa were also recovered by immunoprecipitation from CM-vector with either the anti-Aβ[1–16] or the anti-Aβ[22–35] antibody (Figure 1A). Immunoreactive Aβ bands with higher molecular masses of approximately 8-kDa and 12-kDa were recovered from CM-vector and CM-APP_Swe_ immunoprecipitated with the anti-Aβ[22–35] antibody coupled with probing immunoblots with anti-Aβ[22–35] (Figure 1A, lower panel). Faint immuoreactive Aβ peptide bands with masses of approximately 16-kDa were recovered from CM-APP_Swe_ immunoprecipitated with either anti-Aβ[1–16] or anti-Aβ[22–35] antibody coupled with immunoblots probed with anti-Aβ[1–16] antibody (Figure 1A, top panel). We next measured the concentration of Aβ_1–42_ in CM-vector and CM-APP_Swe_ using a human Aβ_1–42_ ELISA. As expected, the amount of Aβ_1–42_ present in CM-APP_Swe_ is significantly greater than that present in CM-vector (11.7 ± 1.3 and 101.9 ± 3.9 pM Aβ_1–42_ for CM-vector and CM-APP_Swe_, respectively; *p* ≤ 0.05; Figure 1B).

It has been demonstrated previously that N-terminal Aβ antibodies can neutralize soluble synaptotoxic forms of Aβ and inhibit adverse effects of Aβ in an epitope-specific manner (Buttini et al., 2005; Shankar et al., 2008; Jin et al., 2011; Zago et al., 2012). Both anti-Aβ[1–16], an N-terminal-directed antibody, and anti-Aβ[22–35], a mid-region directed antibody, effectively recovered Aβ peptides from CM-APP_Swe_ by immunoprecipitation (Figure 1A). We next compared the ability of anti-Aβ[1–16] and anti-Aβ[22–35] antibodies to neutralize Aβ_1–42_ peptides present in CM-APP_Swe_. To determine this, CM-vector and CM-APP_Swe_ were incubated for 24 h with either anti-HA, anti-Aβ[1–16] or anti-Aβ[22–35] antibody, then Aβ_1–42_ concentrations in antibody-treated media were measured using a human Aβ_1–42_ ELISA that recognizes the N-terminus and C-terminus of Aβ_1–42_. As shown in Figure 1C, incubating CM-APP_Swe_ for 24 h with anti-Aβ[1–16] antibody significantly reduces Aβ_1–42_ concentration in comparison to that found in CM-APP_Swe_ incubated for 24 h with either anti-HA or anti-Aβ[22–35] antibody (97.8 ± 2.5, 18.2 ± 2.8 and 204.5 ± 78 pM Aβ_1–42_ for CM-APP_Swe_ incubated with anti-HA, anti-Aβ[1–16] or anti-Aβ[22–35] antibody, respectively; *p ≤ 0.05). While the anti-Aβ[22–35] antibody bound to and could immunoprecipitate Aβ peptides from CM-APP_Swe_ (Figure 1A), the Aβ_1–42_ concentration was not decreased by this antibody possibly because it does not bind to either the N-terminal or C-terminal amino acids of Aβ_1–42_ that are detected in the ELISA. While not statistically significant, the mean Aβ_1–42_ concentration was higher in CM-APP_Swe_ incubated with the anti-Aβ[22–35] antibody compared to CM-APP_Swe_ incubated with the anti-HA antibody. Aβ_1–42_ levels were not measurably changed in CM-vector incubated with either anti-HA, anti-Aβ[1–16] or anti-Aβ[22–35] antibody (12.1 ± 0.8, 8.0 ± 2.3 and 8.7 ± 1.8 pM Aβ_1–42_, respectively; *p* ≤ 0.05; Figure 1C).

CM-vector and CM-APP_Swe_ used for cell treatments in this study were collected from successive passages of SY5Y-CHT cells transiently expressing either vector or APP_Swe_. The data above represent CM-vector and CM-APP_Swe_ collected from a single passage of SY5Y-CHT cells. Similar Aβ concentrations and Aβ immunoblot profiles between CM-vector and CM-APP_Swe_ collected from successive cell passages were confirmed by Aβ_1–42_ ELISA to measure Aβ_1–42_ concentration in the medium and immunoprecipitation of Aβ to assess the amount and molecular masses of the recovered Aβ peptides.

### Aβ-mediated Decrease in High-Affinity Choline Uptake by CHT Is Attenuated by Aβ N-terminal Antibody

**Figure 2:**
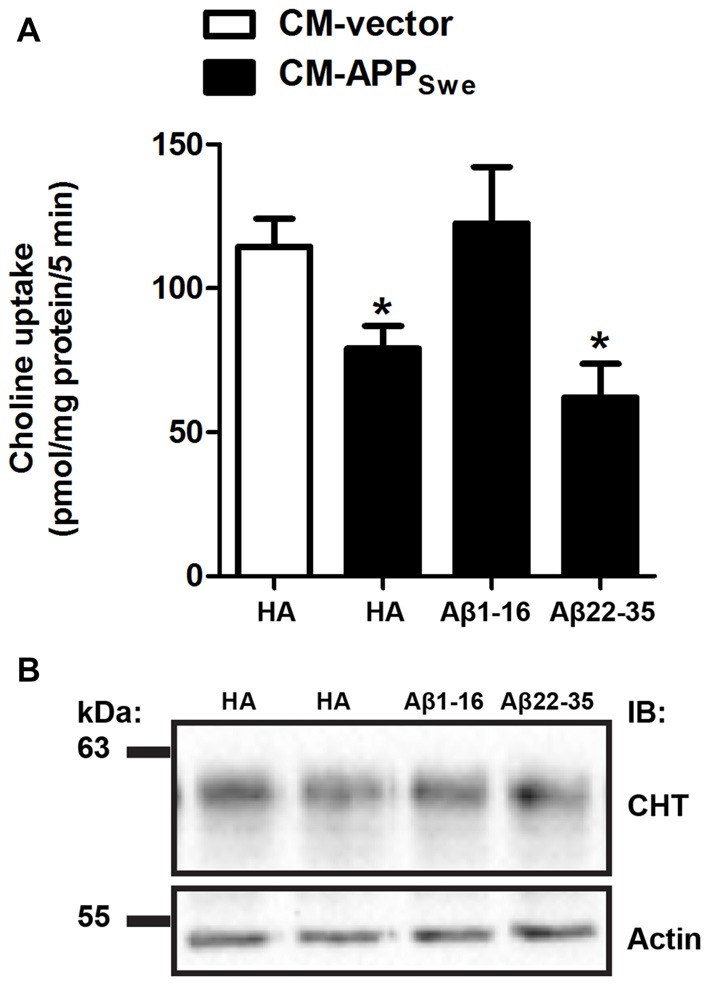
N-terminal Aβ antibody attenuates Aβ-mediated inhibition of [^3^H]-choline uptake activity by choline transporter (CHT). **(A)** CM was collected from SY5Y-CHT cells that had been transiently expressing either vector or APP_Swe_ plasmid DNA for 24 h (CM-vector and CM-APP_Swe_, respectively). CM-vector and CM-APP_Swe_ were incubated with either anti-HA, anti-Aβ[1–16] or anti-Aβ[22–35] antibody for 24 h, added to SY5Y-CHT cells for 24 h, then choline uptake was assayed. CHT activity was significantly reduced in cells treated with CM-APP_Swe_ containing HA and in cells treated with CM-APP_Swe_ containing anti-Aβ[22–35] when compared to cells treated with CM-vector or CM-APP_Swe_ containing anti-Aβ[1–16]. Data are the mean ± SEM of five independent experiments, with statistical analyses performed using a repeated-measures one-way ANOVA with Tukey’s *post hoc* multiple comparisons test (*p ≤ 0.05). **(B)** Representative immunoblots show steady-state total CHT and actin protein levels in total cell lysates from a representative choline uptake experiment.

The N-terminal directed anti-Aβ[1–16] and mid-region directed anti-Aβ[22–35] antibodies both effectively bind Aβ in CM-APP_Swe_ at different epitopes (Figure 1A). The purpose of the next experiment was to compare the effect of neutralizing Aβ in CM-APP_Swe_ with either anti-Aβ[1–16] or anti-Aβ[22–35] antibody on high-affinity choline uptake in SY5Y-CHT cells. To accomplish this, CM-vector was incubated with anti-HA antibody and CM-APP_Swe_ was incubated with either anti-HA, anti-Aβ[1–16] or anti-Aβ[22–35] antibody for 24 h. SY5Y-CHT cells were then incubated with the antibody-treated CM-vector or CM-APP_Swe_, and [^3^H]choline uptake activity was measured. Choline uptake activity is significantly decreased in SY5Y-CHT cells treated with CM-APP_Swe_ containing anti-HA antibody when compared to cells treated with CM-vector containing anti-HA antibody (*p* ≤ 0.05; Figure 2A). Interestingly, this significant decrease in high-affinity choline uptake activity was attenuated by incubation of CM-APP_Swe_ with anti-Aβ[1–16] antibody, but not with anti-Aβ[22–35] antibody (Figure 2A; *p* ≤ 0.05). High-affinity choline uptake activity in SY5Y-CHT cells treated with CM-vector did not differ statistically from that measured in cells treated with CM-APP_Swe_ containing anti-Aβ[1–16] antibody. Total CHT protein levels were equivalent across the treatment groups (Figure 2B).

### Aβ N-terminal Antibody Attenuates Aβ-mediated Loss of CHT Cell Surface Proteins

**Figure 3:**
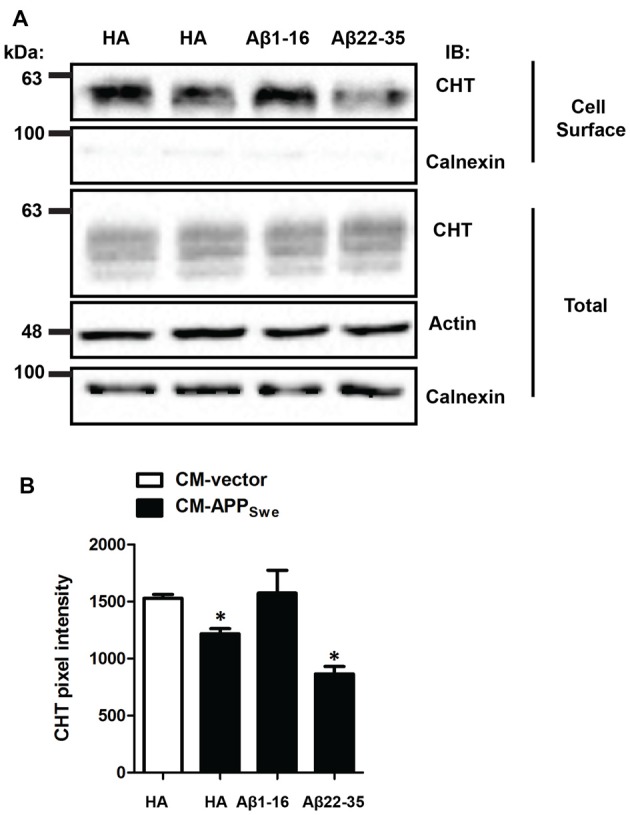
N-terminal Aβ antibody attenuates Aβ-mediated inhibition of CHT plasma membrane levels. **(A)** CM was collected from SY5Y-CHT cells that had been transiently expressing either vector or APP_Swe_ plasmid DNA for 24 h (CM-vector and CM-APP_Swe_, respectively). CM-vector and CM-APP_Swe_ were incubated with either anti-HA, anti-Aβ[1–16] or anti-Aβ[22–35] antibody for 24 h and then added to SY5Y-CHT cells for 24 h. Cells were then washed and placed on ice, and then plasma membrane proteins were biotinylated. Biotinylated proteins were captured using NeutrAvidin agarose, then proteins were solubilized and separated by SDS-PAGE. PVDF membranes were processed by immunoblotting with antibodies recognizing CHT, actin and calnexin. Representative immunoblots show steady-state CHT, actin and calnexin protein levels in total cell lysates (3 lower panels). The top panels illustrate cell surface (biotinylated) CHT and calnexin proteins. The immunoblots shown are representative of data obtained from five independent experiments. **(B)** Analysis of cell surface CHT protein bands by densitometry reveals that the level of CHT protein at the cell surface is significantly reduced in cells treated with CM-APP_Swe_ containing HA and in cells treated with CM-APP_Swe_ containing anti-Aβ[22–35] antibody when compared to cells treated with CM-vector or CM-APP_Swe_ containing anti-Aβ[1–16] antibody. Data are the mean ± SEM of five independent experiments, with statistical analyses performed using a repeated-measures one-way ANOVA with Tukey’s *post hoc* multiple comparisons test (*p ≤ 0.05).

Since the anti-Aβ[1–16] antibody neutralizes soluble Aβ peptides and prevents the decrease in high-affinity choline uptake seen in SY5Y-CHT cells treated with CM-APP_Swe_, we predicted that binding of anti-Aβ[1–16] antibody to Aβ would similarly protect against Aβ-mediated loss of CHT cell surface expression. Thus, we performed cell surface protein biotinylation experiments using SY5Y-CHT cells treated for 24 h with CM-vector that had been incubated with anti-HA antibody or with CM-APP_Swe_ that had been incubated with either anti-HA, anti-Aβ[1–16] or anti-Aβ[22–35] antibody for 24 h. Plasma membrane proteins were then biotinylated using membrane impermeable sulfo-NHS-biotin at 4°C. Representative immunoblots in Figure 3A show the cell surface levels of (biotinylated) CHT and calnexin proteins and the total amount of CHT, calnexin and actin proteins in cell lysates. Quantitative analysis of cell surface CHT protein immunoblots (top panel) reveals that CHT cell surface levels are significantly lower in SY5Y-CHT cells treated with CM-APP_Swe_ containing either anti-HA or anti-Aβ[22–35] antibody, but not in SY5Y-CHT cells treated with CM-APP_Swe_ containing anti-Aβ[1–16] antibody when compared to CM-vector treated SY5Y-CHT cells (Figure 3B; *p* ≤ 0.05). Total CHT protein levels are equal between cells treated with either CM-vector or CM-APP_Swe_ incubated with either anti-HA, anti-Aβ[1–16] or anti-Aβ[22–35] antibody (middle panel). CHT cell surface levels did not differ between SY5Y-CHT cells treated with CM-vector and cells treated with CM-APP_Swe_ containing anti-Aβ[1–16] (Figure 3A, lanes 1 and 3, respectively). The absence of calnexin immunoreactivity in biotinylated cell surface fractions and its presence in total cell lysate fractions confirmed the isolation of cell surface proteins.

### Aβ-mediated Decrease in CHT Co-Localization with Early Endosome EEA1 Is Attenuated by N-terminal Aβ Antibody

**Figure 4:**
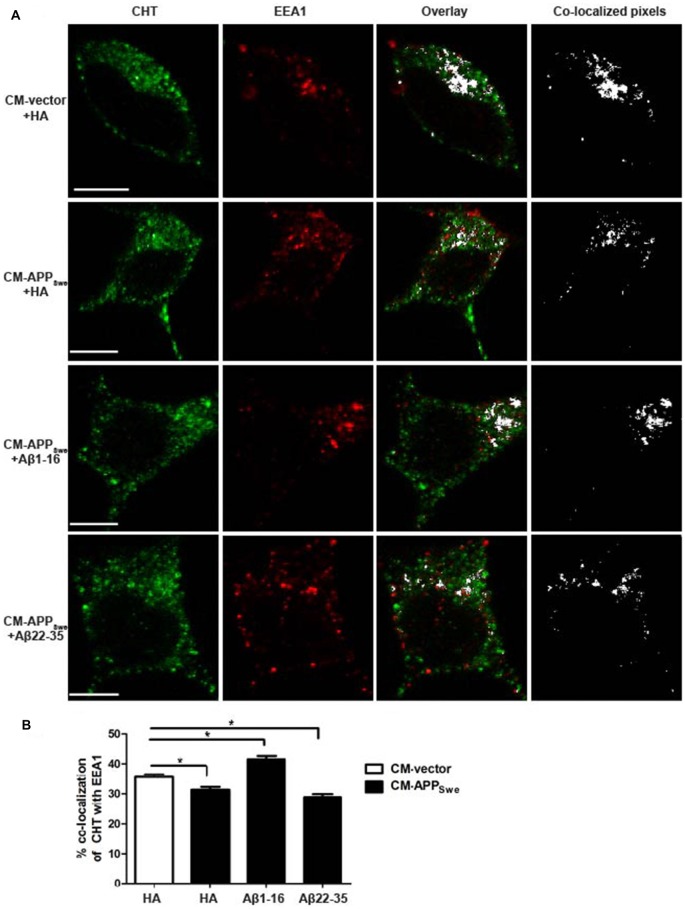
Subcellular distribution of CHT with early endosome marker EEA1 in SY5Y-CHT cells treated with CM derived from SY5Y-CHT cells expressing APP_Swe_. **(A)** CM was collected from SY5Y-CHT cells that had been transiently expressing either vector or APP_Swe_ plasmid DNA for 24 h (CM-vector and CM-APP_Swe_, respectively). CM-vector and CM-APP_Swe_ were incubated with either anti-HA antibody, anti-Aβ[1–16] or anti-Aβ[22–35] antibody for 24 h and then added to SY5Y-CHT cells for 24 h and then cells were formalin-fixed. Confocal images show the distribution of AlexaFluor 488-labeled CHT (green) and AlexaFluor 555-labeled EEA1 (red). Co-localized pixels were identified in the co-localization channel and are shown as white in the right co-localized pixels panels. Scale bars, 5 μM. **(B)** CHT and EEA1 pixels that were determined to be co-localized in the co-localization channel were quantified using Imaris software. Data are expressed as the mean ± SEM for a minimum of 15 cells per transfection group from five independent experiments, and were analyzed by one-way ANOVA followed by Tukey’s *post hoc* multiple comparisons test (*p ≤ 0.05).

Plasma membrane CHT levels are maintained by constitutive recycling of CHT proteins between endocytic compartments and the cell surface, thereby regulating choline uptake activity. CHT proteins internalize rapidly from the plasma membrane to early endosomes by a clathrin-mediated process, thus we investigated whether Aβ present in CM-APP_Swe_ alters CHT localization to early endosomes. To this end, we used confocal microscopy to visualize the co-localization of CHT and early endosome maker EEA1 in SY5Y-CHT cells treated with either CM-vector containing anti-HA antibody or CM-APP_Swe_ containing either anti-HA, anti-Aβ[1–16] or anti-Aβ[22–35] antibody. To assess the extent of co-localization between CHT and EEA1, we used a quantitative approach using Imaris software (Bitplane) to set threshold fluorescence intensities that filter the brightest 2% of pixels of CHT (shown in green) that also fall within the brightest 2% of pixels of EEA1 (shown in red). This analysis is described further in Hutcheon et al. (2000) and Lorenzen et al. (2010). The co-localized pixels are identified in a separate co-localization channel and shown as white in the right overlay panels of Figure 4A. As shown in Figure 4B, analysis of the quantified pixels revealed that CHT co-localizes significantly less with EEA1 in cells treated with CM-APP_Swe_ incubated with either anti-HA or anti-Aβ[22–35] antibody (31.3 ± 1.0% and 28.9 ± 1.0%, respectively) compared to cells treated with either CM-vector or CM-APP_Swe_ containing with anti-Aβ[1–16] antibody (35.6 ± 1.0% and 41.5 ± 1.1%, respectively). Unexpectedly, CHT co-localizes significantly more with EEA1 in cells treated with CM-APP_Swe_ containing anti-Aβ[1–16] compared to cells treated to CM-vector treated cells. The reason for this is unknown, but could be due to low physiological levels of Aβ present in CM-vector and not present in CM-APP_Swe_ containing anti-Aβ[1–16], thereby regulating CHT recycling between early endosomes and the cell surface.

### Aβ-mediated Decrease in CHT Co-Localization with Lysosome Marker LAMP-1 Is Attenuated by N-terminal Aβ Antibody

**Figure 5:**
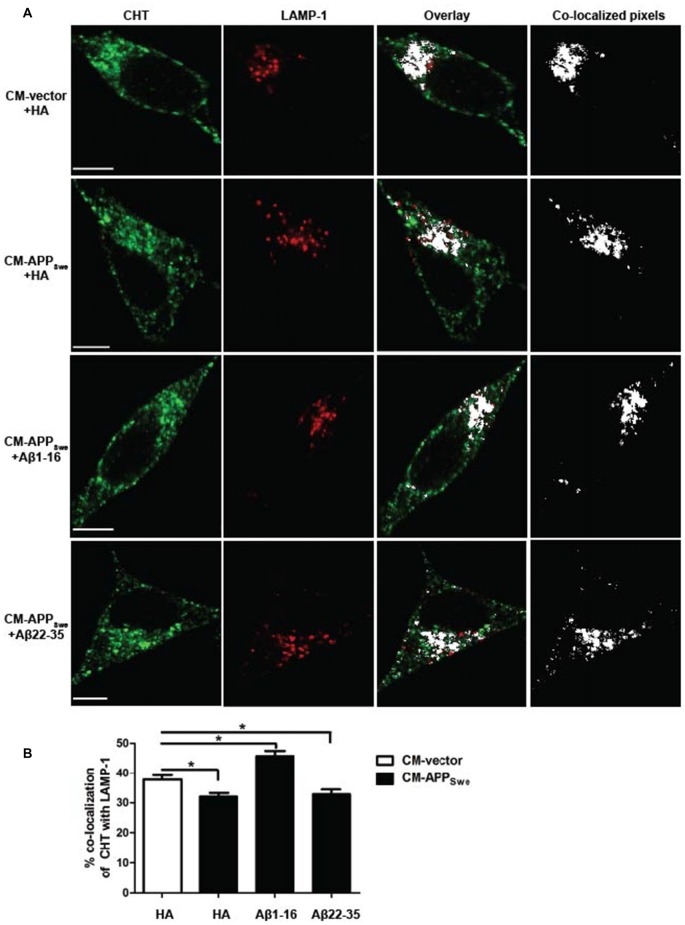
Subcellular distribution of CHT with lysosome marker LAMP-1 in SY5Y-CHT cells treated with CM derived from SY5Y-CHT cells expressing APP_Swe_. **(A)** CM was collected from SY5Y-CHT cells that had been transiently expressing either vector or APP_Swe_ plasmid DNA for 24 h (CM-vector and CM-APP_Swe_, respectively). CM-vector and CM-APP_Swe_ were incubated with either anti-HA antibody, anti-Aβ[1–16] or anti-Aβ[22–35] antibody for 24 h and then added to SY5Y-CHT cells for 24 h and then cells were formalin-fixed. Confocal images show the distribution of AlexaFluor 647-labeled CHT (green) and AlexaFluor 488-labeled LAMP-1 (red). Co-localized pixels were identified in the co-localization channel and are shown as white in the right co-localized pixels panels. Scale bars, 5 μM. **(B)** CHT and LAMP-1 pixels that were determined to be co-localized in the co-localization channel were quantified using Imaris software. Data are expressed as the mean ± SEM for a minimum of 18 cells per transfection group from four independent experiments, and were analyzed by one-way ANOVA followed by Tukey’s *post hoc* multiple comparisons test (*p ≤ 0.05).

CHT proteins internalize to early endosomes from the plasma membrane and either recycle back to the cell surface or move through late endosomes to lysosomes for degradation (Cuddy et al., 2012). Our results reveal that Aβ causes a significant decrease in CHT activity and cell surface expression that corresponds with a significant loss of CHT proteins from early endosomes. Thus, we hypothesize that the Aβ-mediated loss of CHT proteins from early endosomes, and the corresponding decrease in CHT cell surface expression and activity, could be related to an increased movement of CHT to lysosomes for degradation. We next used confocal microscopy to visualize the co-localization of CHT and lysosome marker LAMP-1 in SY5Y-CHT cells treated with either CM-vector containing anti-HA antibody or CM-APP_Swe_ containing either anti-HA, anti-Aβ[1–16] or anti-Aβ[22–35] antibody. To assess the extent of co-localization between CHT and LAMP-1, we used the quantitative approach described above to set threshold fluorescence intensities that filter the brightest 2% of pixels of CHT (shown in green) that also fall within the brightest 2% of pixels of LAMP-1 (shown in red). The co-localized pixels are identified in a separate co-localization channel and shown as white in the right overlay panels of Figure 5A. As shown in Figure 5B, analysis of the quantified pixels revealed that CHT co-localizes significantly less with LAMP-1 in cells treated with CM-APP_Swe_ incubated with either anti-HA or anti-Aβ[22–35] antibody (32 ± 1.2% and 33 ± 1.6%, respectively) compared to cells treated with either CM-vector or CM-APP_Swe_ containing anti-Aβ[1–16] antibody (38 ± 1.4% and 45 ± 1.6%, respectively). Consistent with our observation that CHT co-localizes more with EEA1 in cells treated with CM-APP_Swe_ containing anti-Aβ[1–16] compared to cells treated to CM-vector treated cells, CHT also co-localizes significantly more with LAMP-1 in cells treated with CM-APP_Swe_ containing anti-Aβ[1–16] compared to cells treated to CM-vector treated cells.

### Lysosome Inhibitor Bafilomycin A1 Prevents Aβ-mediated Decrease in CHT Co-Localization with LAMP-1

**Figure 6:**
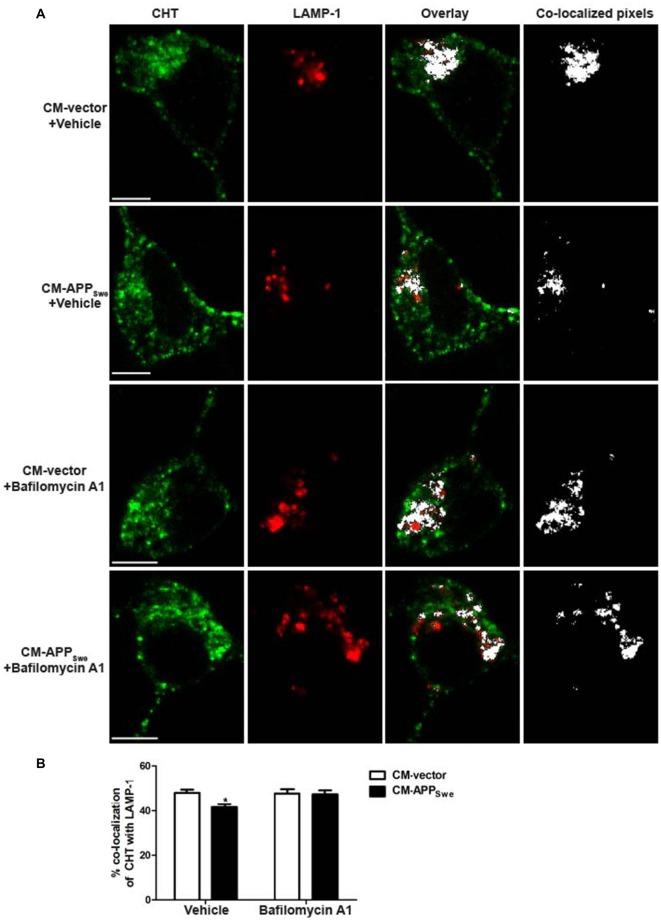
Subcellular distribution of CHT with lysosome marker LAMP-1 in SY5Y-CHT cells treated with CM derived from SY5Y-CHT cells expressing APP_Swe_ containing bafilomycin A1 (BafA_1_). **(A)** CM was collected from SY5Y-CHT cells that had been transiently expressing either vector or APP_Swe_ plasmid DNA for 24 h (CM-vector and CM-APP_Swe_, respectively). CM-vector and CM-APP_Swe_ containing either vehicle (DMSO) or 20 nM BafA_1_ were added to SY5Y-CHT cells for 24 h and then cells were formalin-fixed. Confocal images show the distribution of AlexaFluor 647-labeled CHT (green) and AlexaFluor 488-labeled LAMP-1 (red). Co-localized pixels were identified in the co-localization channel and are shown as white in the right co-localized pixels panels. Scale bars, 5 μM. **(B)** CHT and LAMP-1 pixels that were determined to be co-localized in the co-localization channel were quantified using Imaris software. Data are expressed as the mean ± SEM for a minimum of 15 cells per transfection group from four independent experiments, and were analyzed by two-way ANOVA followed by Tukey’s *post hoc* multiple comparisons test (*p ≤ 0.05).

Aβ peptides present in CM-APP_Swe_ decrease the amount of CHT localizing to both early endosomes and lysosomes (Figures 4, 5, respectively). To investigate whether the Aβ-mediated loss of CHT proteins from early endosomes and lysosomes is related to increased CHT degradation by the lysosome, we blocked proteolytic activity of the lysosome pharmacologically using BafA_1_ (which blocks acidification of the lysosome), and measured CHT co-localization with LAMP-1 by confocal microscopy. In this experiment, SY5Y-CHT cells were treated with either CM-vector or CM-APP_Swe_ containing either vehicle or BafA_1_ and the extent of co-localization between CHT and LAMP-1 was assessed using the quantitative approach described above. Co-localization between CHT and LAMP-1 was observed in cells treated with either CM-vector or CM-APP_Swe_ containing either vehicle or BafA_1_ (Figure 6A). Co-localized pixels of CHT and LAMP-1 appear white in the overlay panels and co-localized pixels panels of these images. As shown in Figure 6B, and consistent with our findings above (Figure 5), analysis of the quantified pixels revealed that CHT co-localizes significantly less with LAMP-1 in cells treated with CM-APP_Swe_ containing vehicle compared to cells treated with CM-vector containing vehicle (41.6 ± 1.5% and 48.1 ± 1.4%, respectively). Importantly, treatment of cells with CM-APP_Swe_ containing BafA_1_ attenuated the effect of CM-APP_Swe_, with co-localization of CHT with LAMP-1 not differing significantly between these treatment groups (47.4 ± 1.7% and 47.1 ± 1.9%, respectively).

### Lysosome Inhibitor Bafilomycin A_1_ Prevents Aβ-mediated Decrease in CHT Cell Surface Expression, but Not CHT Activity

We investigated whether Aβ inhibits CHT function by increasing lysosomal degradation of CHT proteins by blocking proteolytic activity of lysosomes using the inhibitor BafA_1_, then measuring CHT cell surface expression and activity using cell surface protein biotinylation and choline uptake assays, respectively.

**Figure 7:**
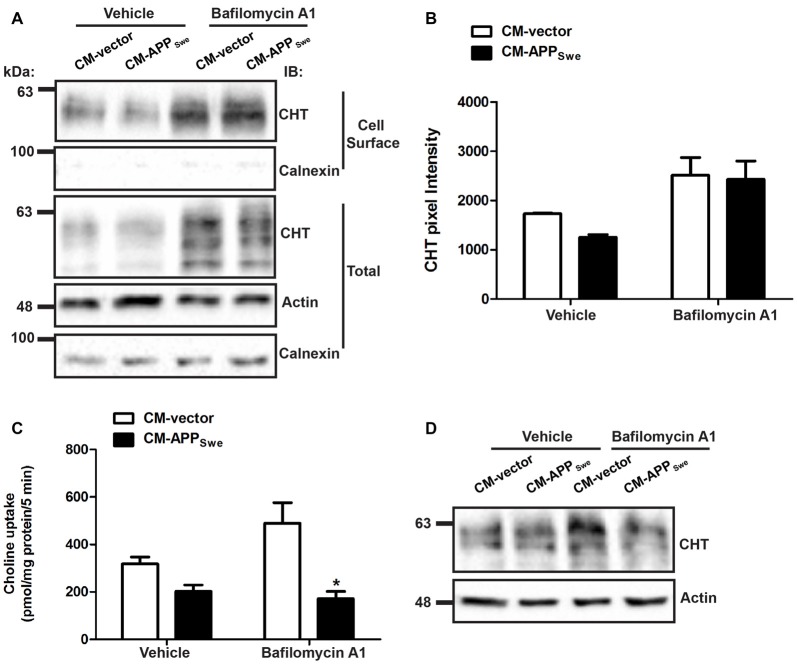
BafA_1_ blocks an Aβ-mediated decrease in CHT cell surface expression but not CHT activity. **(A)** CM was collected from SY5Y-CHT cells that had been transiently expressing either vector or APP_Swe_ plasmid DNA for 24 h (CM-vector and CM-APP_Swe_, respectively). CM-vector and CM-APP_Swe_ containing either vehicle (DMSO) or 20 nM BafA_1_ were added to SY5Y-CHT cells for 24 h. Cells were then washed and placed on ice, and then plasma membrane proteins were biotinylated. Representative immunoblots show steady-state CHT, actin and calnexin protein levels in total cell lysates (3 lower panels). The top panels illustrate cell surface (biotinylated) CHT and calnexin proteins. The immunoblots shown are representative of data obtained from five independent experiments. **(B)** Analysis of cell surface CHT protein bands by densitometry reveals that the level of CHT protein at the cell surface is reduced in cells treated with CM-APP_Swe_ containing vehicle compared to cells treated with CM-vector containing vehicle. No differences in the level of CHT protein at the cell surface were observed in cells treated with CM-vector containing BafA_1_ compared to cells treated with CM-APP_Swe_ containing BafA_1_. Data are the mean ± SEM of five independent experiments, with statistical analyses performed using a two-way ANOVA with Bonferroni *post hoc* multiple comparisons test (*p ≤ 0.05). **(C)** CM-vector and CM-APP_Swe_ containing either vehicle or 20 nM BafA_1_ were added to SY5Y-CHT cells for 24 h, and then choline uptake was assayed. CHT activity was reduced in cells treated with CM-APP_Swe_ containing vehicle compared to cells treated with CM-vector containing vehicle. CHT activity was significantly reduced in cells treated with CM-APP_Swe_ containing BafA_1_ when compared to cells treated with CM-vector containing BafA_1_. Data are the mean ± SEM of five independent experiments, with statistical analyses performed using a two-way ANOVA with Bonferroni *post hoc* multiple comparisons test (*p ≤ 0.05). **(D)** Representative immunoblots show steady-state total CHT and actin protein levels in total cell lysates from a representative choline uptake experiment.

To determine the effect of lysosome inhibition on CHT cell surface expression, SY5Y-CHT cells were treated with either CM-vector or CM-APP_Swe_ containing either vehicle or BafA_1_ for 24 h. Plasma membrane proteins were then biotinylated using membrane impermeable sulfo-NHS-biotin at 4°C. Representative immunoblots in Figure 7A show the level of cell surface (biotinylated) CHT and calnexin proteins and the amount of total CHT, calnexin and actin protein. Total CHT protein levels (middle panel) are equal between cells treated with either CM-vector or CM-APP_Swe_ containing vehicle, while total CHT protein levels are substantially increased in cells treated with CM containing BafA_1_ compared to cells treated with CM containing vehicle. Quantitative analysis was carried out on immunoblots of biotinylated cell surface CHT protein levels (top panel). Statistical analysis by two-way ANOVA reveals no interaction between media treatment and drug (BafA_1_) treatment of cells (*P* = 0.46), but there is a statistically significant difference related to BafA_1_ treatment of cells (*P* = 0.0018) with this lysosome inhibitor attenuating the decrease in cell surface CHT protein levels observed in cells treated with CM-APP_Swe_ containing vehicle. In SY5Y-CHT cells treated with CM-APP_Swe_ containing vehicle, cell surface CHT protein levels are decreased by 28% when compared to cells treated with CM-vector containing vehicle (Figure 7B); this is comparable to the statistically significant 22% decrease in CHT cell surface levels for the same treatment groups shown in Figure 3. BafA_1_ treatment attenuated the effect of CM-APP_Swe_, with CHT cell surface levels being the same for cells treated with CM-vector and CM-APP_Swe_. The absence of calnexin immunoreactivity in biotinlyated cell surface fractions and presence in total cell lysate fractions confirmed the isolation of cell surface proteins.

Since BafA_1_ prevents the Aβ-mediated decrease in CHT cell surface expression in SY5Y-CHT cells treated with CM-APP_Swe_, we predicted that BafA_1_ would also block an Aβ-mediated decrease in high-affinity choline uptake activity. To test this, cells were treated with either CM-vector or CM-APP_Swe_ containing either vehicle or BafA_1_ for 24 h, then [^3^H]choline uptake activity was measured (Figure 7C). Statistical analysis by two-way ANOVA revealed no interaction between media treatment and drug (BafA_1_) treatment (*P* = 0.06), and while there was no difference related to BafA_1_ treatment (*P* = 0.18), a significant difference was found for media treatment (*P* = 0.0005). In SY5Y-CHT cells treated with CM-APP_Swe_ containing vehicle, choline uptake activity is decreased by 37% when compared to cells treated with CM-vector containing vehicle (Figure 7C); this is comparable to the statistically significant 31% decrease in choline uptake activity for the same treatment groups shown in Figure 2. Interestingly, high-affinity choline uptake activity was not increased in SY5Y-CHT cells treated with CM-APP_Swe_ containing BafA_1_ when compared to vehicle. Figure 7D is a representative experiment showing that total sample CHT and actin protein levels were equivalent across the treatment groups.

## Discussion

We investigated the effect of Aβ peptides present in CM collected from APP_Swe_-expressing cells (CM-APP_Swe_) on CHT trafficking and activity. APP containing the Swedish mutation that causes familial AD undergoes high-efficiency amyloidogenic cleavage, increasing Aβ production by 10-fold (Haass et al., 1995; Thinakaran et al., 1996). We found recently that, when compared to wild-type APP, expression of APP_Swe_ in neural cells decreases CHT function by a mechanism that was related to increased APP_Swe_ processing (Cuddy et al., 2015). We now make the novel observation that treatment of neural cells with CM-APP_Swe_ decreases CHT co-localization with the early endosome marker EEA1 and lysosome marker LAMP-1. Moreover, we found that the lysosome inhibitor BafA_1_ attenuates Aβ-mediated decreases in CHT cell surface levels. However, lysosome inhibition did not block the effect of Aβ on CHT activity. Finally, inhibition of CHT function by Aβ peptides was blocked by an antibody directed at the N-terminal amino acids 1–16 of Aβ (anti-Aβ[1–16]), but not by an antibody directed at the mid-region amino acids 22–35 of Aβ (anti-Aβ[22–35]).

Aβ peptides assemble into soluble oligomers, protofibrils and fibrils that accumulate in the brains of AD subjects. It was considered initially that fibrillar Aβ was responsible for cholinergic dysfunction seen early in AD, but cognitive impairment correlates better with the level of soluble Aβ oligomers than with fibril deposition, suggesting that Aβ oligomers are the more toxic Aβ species (Shankar et al., 2008; Li et al., 2009). While soluble Aβ peptides alter neuron function, Aβ preparations from different sources including synthetic and cell-derived Aβ peptides can differ in toxicity and potency. For example, soluble Aβ produced by mutant APP-expressing 7PA2 cells is 100 times more potent than synthetic Aβ in producing errors in a cognitive function assay in rats (Reed et al., 2012). Synthetic preparations of soluble Aβ can inhibit high-affinity choline uptake (Kar et al., 1998; Kristofiková et al., 2001; Parikh et al., 2014), thus we tested the effect of Aβ peptides released from cells on CHT function. Our data reveal that low pM concentrations of soluble Aβ present in CM-APP_Swe_ significantly inhibit choline uptake activity in SY5Y-CHT cells. These findings agree with previous reports that chronic and acute treatment with Aβ peptides in the fM to μM concentration range significantly inhibit choline uptake in both *in vitro* and *in vivo* models (Kar et al., 1998; Kristofikova et al., 2013; Parikh et al., 2014). Together, these data show that CHT proteins are highly sensitive to Aβ peptides, suggesting that early changes in AD brain that cause small shifts in Aβ generation could have a large impact on CHT activity and cholinergic transmission.

Little is known about the mechanism by which Aβ peptides impair high-affinity choline uptake. We show that this Aβ-mediated inhibition corresponds to a significant decrease in CHT cell surface levels in SY5Y-CHT cells and predicted that Aβ promotes CHT internalization, which has also been observed for N-methyl-D aspartate (NMDA) and α-amino-3-hydroxy-5-methyl-4-isoxazole-propionic acid (AMPA) glutamate receptors (Hsieh et al., 2006; Dewachter et al., 2009). We found significantly decreased CHT localization in early endosomes and lysosomes in SY5Y-CHT cells treated for 24 h with CM-APP_Swe_. Previous data reveal that CHT proteins internalize into early endosomes from the plasma membrane by a dynamin-dependent, clathrin-mediated mechanism with a t_1/2_ of about 15 min (Ribeiro et al., 2005; Cuddy et al., 2012). CHT proteins then either recycle back to the plasma membrane, or move to late endosomes and lysosomes within about 30 min after internalization where they normally undergo degradation (Cuddy et al., 2012). Oxidative-nitrosative stress can result in enhanced ubiquitination of CHT proteins, with some transporters directed to proteosomal degradation (Cuddy et al., 2012). One explanation for our findings is that Aβ increases CHT protein internalization and movement to lysosomes where they are degraded, resulting in fewer CHT proteins at the cell surface, in early endosomes and in lysosomes at 24 h. While total CHT protein levels in cells were unchanged, this is likely due to the relatively small proportion of the total cellular CHT proteins that are present at the plasma membrane and in the recycling pool that feeds CHT proteins to the cell surface. In both cultured cells and rat brain nerve endings, only 15% of total CHT proteins are contained within this recycling pool and at the cell surface, with the majority being found in other intracellular vesicular compartments (Ferguson et al., 2003; Ribeiro et al., 2005). Thus, an Aβ-mediated decrease in the pool of CHT proteins present at the cell surface may not be detected when measuring total cellular CHT levels.

To investigate whether exposure of cells to CM-APP_Swe_ containing Aβ peptides increases lysosomal degradation of CHT, we blocked proteolytic activity of lysosomes using BafA_1_ and measured the effect on subcellular localization and function of CHT. In support of our hypothesis that Aβ increases lysosomal degradation of CHT, BafA_1_ attenuated the CM-APP_Swe_-mediated decrease in CHT proteins in lysosomes and also blocked the decrease in CHT cell surface levels. Inhibition of lysosomal degradation of CHT by BafA_1_ was confirmed by an increase in total and cell surface CHT protein levels. The increase in cell surface levels of CHT protein in BafA1-treated cells grown in CM-APP_Swe_ medium may be due to less CHT being degraded and actively recycling back to the plasma membrane. Interestingly, BafA_1_ treatment attenuated the effect of CM-APP_Swe_ on CHT cell surface levels, but not on CHT activity. An explanation for this finding is that the conformational state of CHT required for solute binding or solute translocation may be altered by Aβ, thereby impairing the function of transporters that are retained at the cell surface. However, the underlying mechanisms for this are unknown. A direct interaction between Aβ and CHT has been reported, but the mechanistic impact of this interaction was not investigated (Bales et al., 2006). It is possible that Aβ interacts with CHT proteins at a solute recognition site and prevents solute binding, as has been observed for both the glutamate and glycine agonist recognition sites of the NMDA receptor (Cowburn et al., 1997). Aβ could also affect CHT function indirectly by its effects on the lipid bilayer. Aβ oligomers bind principally to membrane lipids and secondarily interrupt the structure and function of several synaptic transmembrane transporters and channels. This mechanism is consistent with the effect of soluble Aβ on glutamate uptake by isolated synaptosomes *in vitro* (Li et al., 2009). We showed previously that CHT proteins are concentrated in lipid rafts and disrupting rafts significantly alters CHT activity and solute binding affinity (Cuddy et al., 2014). Moreover, disruption of lipid rafts enhances the Aβ-mediated inhibition of CHT activity (Kristofikova et al., 2013). Together, these data indicate that lipid rafts maintain CHT in a functional conformation required for either solute binding or translocation and this may be altered by Aβ.

To confirm that CHT inhibition in CM-APP_Swe_ treated cells was due to Aβ peptides and not to other proteolytic fragments of APP, we tested the effects of neutralizing Aβ peptides in CM-APP_Swe_ using anti-Aβ antibodies. Several antibodies targeting different epitopes of Aβ have been developed, with some being evaluated clinically for the treatment of mild to moderate AD. Most studies have focused on N-terminal antibodies, based on results obtained from active immunization strategies using B-cell epitopes of Aβ located in the N-terminus of the peptide (Schneeberger et al., 2009; Spencer and Masliah, 2014). Bapineuzumab, an N-terminal Aβ antibody targeted at residues 1–5 of Aβ was the first immunotherapy in clinical trials, but showed only minimal cognitive benefits (Tayeb et al., 2013; Salloway et al., 2014). Solanezumab, a mid-region-directed Aβ antibody recognizing residues 13–28 of Aβ showed a significant 33% reduction in rate of decline in patients with mild AD (Doody et al., 2014). In preclinical studies, N-terminal Aβ antibodies can neutralize and block the deposition of toxic species of Aβ peptides and show beneficial effects in various AD mouse models by preventing synaptic degeneration and reversing memory deficits in object recognition and Morris water maze (Dodart et al., 2002; Bard et al., 2003; Buttini et al., 2005). In the present study, we compared the effect of neutralizing CM-APP_Swe_ with either N-terminal Aβ antibody 6E10 that recognizes amino acids 1–16 (anti-Aβ[1–16]) or mid-region Aβ antibody recognizing amino acids 22–35 (anti-Aβ[22–35]) on CHT function. Our data reveal that anti-Aβ[1–16], but not anti-Aβ[22–35], blocked inhibition of CHT function by Aβ present in CM-APP_Swe_.

Our data suggest that the site at which Aβ impacts CHT function lies within the N-terminal amino acids 1–16 of Aβ peptide. While this observation is in agreement with evidence showing a neuroprotective effect of N-terminal Aβ antibodies, other studies have suggested that the mid-region of Aβ is responsible for mediating CHT dysfunction. Structure-activity analysis shows that the non-aggregated synthetic Aβ fragments Aβ_1–42_, Aβ_1–40_, Aβ_1–28_ and Aβ_25–35_ inhibit high-affinity choline uptake and ACh release from rat hippocampal slices similarly, suggesting that Aβ peptide residues 25–28 are required for these effects (Kar et al., 1998). In a separate study, the anti-Aβ antibody m266 that recognizes amino acids 13–28 of Aβ restored hippocampal ACh release and high-affinity choline uptake and reduced impaired habituation learning in PDAPP mice, but the anti-Aβ antibody 3D6 directed at amino acids 1–5 of Aβ was without effect (Bales et al., 2006). The differences in these findings could be explained by differences in the mechanism by which low concentrations of naturally-produced soluble Aβ used in the present study inhibit CHT activity, compared to non-aggregated synthetic peptides of Aβ or high concentrations of fibrillar Aβ present in the brains of PDAPP mice. Moreover, anti-Aβ antibodies display epitope specificities in binding and prevention of formation of distinct Aβ species, which could differentially affect CHT function. For example, it has been proposed that the anti-Aβ m266 antibody may inhibit Aβ fibril formation, but have no effect on Aβ oligomerization (Legleiter et al., 2004). Interestingly, it was recently shown that 3D6, the murine form of N-terminal Aβ antibody Bapineuzumab, binds to soluble assemblies of Aβ_1–42_ and prevents functional consequences in hippocampal neurons including changes in glutamate AMPA receptor trafficking (Zago et al., 2012). We did not investigate whether differences exist in the ability of anti-Aβ[1–16] and anti-Aβ[22–35] to bind to or prevent the formation of different Aβ assemblies in CM-APP_Swe_. However, anti-Aβ[1–16] and anti-Aβ[22–35] appear to immunoprecipitate different higher molecular weight Aβ species from CM-APP_Swe_ with anti-Aβ[1–16] isolating higher molecular weight 16-kDa Aβ species while anti-Aβ[22–35] isolated 8-kDa and 12-kDa Aβ-species.

In conclusion, we report novel observations regarding the regulation of CHT function by Aβ. We reveal for the first time that naturally produced soluble forms of Aβ increase lysosomal degradation of CHT, but make the critical observation that while blocking this pathway does restore cell surface CHT protein levels it does not attenuate the effect of Aβ on CHT activity. This suggests that Aβ may result in altered protein-protein interactions or post-translational modification of CHT, thereby increasing its movement to the lysosome. It may also cause important effects on the conformational state of CHT required for choline uptake activity, either directly or indirectly through effects on the lipid bilayer. Moreover, we show that an N-terminal Aβ antibody binds with soluble forms of Aβ and attenuates the effect of Aβ on CHT activity and trafficking. Interestingly, a mid-region Aβ antibody did not alter Aβ effects on CHT, indicating that a specific N-terminal Aβ epitope or conformation of soluble Aβ may impair CHT activity. Together, our data suggest that therapeutic strategies that prevent Aβ binding to CHT could be more effective in the treatment or prevention of AD than strategies designed to promote CHT cell surface expression.

## Author Contributions

LKC performed all experiments except for cell imaging, and CS performed cell imaging. LKC, CS and RJR designed the experiments and were involved in analysis and interpretations of data, and in drafting, revision and critical analysis of the manuscript. SHP was involved in analysis and interpretations of data, drafting, revision and critical analysis of the manuscript. All authors agree to be accountable for all aspects of the work and ensure that all questions related to accuracy or integrity of the article have been appropriately investigated and resolved, and give final approval for the version to be published.

## Conflict of Interest Statement

The authors declare that the research was conducted in the absence of any commercial or financial relationships that could be construed as a potential conflict of interest.

## Abbreviations

Aβ: β-amyloid
ACh: acetylcholine
AD: Alzheimer’s disease
ANOVA: analysis of variance
APP: amyloid precursor protein
BafA_1_: bafilomycin A1
CHT: high-affinity choline transporter
HC-3: hemicholinium-3
KRH: Krebs-Ringer-HEPES.

## References

[B92] Auld D. S. Kar S. Quirion R. (1998). β-amyloid peptides as direct cholinergic neuromodulators: a missing link? Trends Neurosci. 21, 43–49. 10.1016/S0166-2236(97)01144-2 9464686

[B1] Bales K. R. Tzavara E. T. Wu S. Wade M. R. Bymaster F. P. Paul S. M.. (2006). Cholinergic dysfunction in a mouse models of Alzheimer disase is reversed by an anti-Aβ antibody. J. Clin. Invest. 116, 825–832. 10.1172/jci27120 16498501 PMC1378188

[B2] Bard F. Barbour R. Cannon C. Carretto R. Fox M. Games D.. (2003). Epitope and isotype specificities of antibodies to β-amyloid peptide for protection against Alzheimer’s disease-like neuropathology. Proc. Natl. Acad. Sci. U S A 100, 2023–2028. 10.1073/pnas.0436286100 12566568 PMC149952

[B3] Bard F. Cannon C. Barbour R. Burke R. L. Games D. Grajeda H.. (2000). Peripherally administered antibodies against amyloid β peptide enter the central nervous system and reduce pathology in a mouse model of Alzheimer disease. Nat. Med. 6, 916–919. 10.1038/78682 10932230

[B4] Black S. A. G. Rylett R. J. (2012). Choline transporter CHT regulation and function in cholinergic neurons. Cent. Nerv. Syst. Agents Med. Chem. 12, 114–121. 10.2174/187152412800792724 22483273

[B5] Buttini M. Masliah E. Barbour R. Grajeda H. Motter R. Johnson-Wood K.. (2005). β amyloid immunotherapy prevents synaptic degeneration in a mouse model of Alzheimer’s disease. J. Neurosci. 25, 9096–9101. 10.1523/JNEUROSCI.1697-05.2005 16207868 PMC6725749

[B6] Chen X. Lin R. Chang L. Xu S. Wei X. Zhang J.. (2013). Enhancement of long-term depression by soluble amyloid β protein in rat hippocampus is mediated by metabotropic glutamate receptor and involves activation of p38MAPK, STEP and caspase-3. Neuroscience 253, 435–443. 10.1016/j.neuroscience.2013.08.054 24012839

[B7] Cowburn R. F. Wiehager B. Trief E. Li-Li M. Sundström E. (1997). Effects of β-amyloid-(25–35) peptides on radioligand binding to excitatory amino acid receptors and voltage-dependent calcium channels: evidence for a selective affinity for the glutamate and glycine recognition sites of the NMDA receptor. Neurochem. Res. 22, 1437–1442. 10.1023/A:1021942109490 9357007

[B8] Cuddy L. K. Gordon A. C. Black S. A. G. Jaworski E. Ferguson S. S. G. Rylett R. J. (2012). Peroxynitrite donor SIN-1 alters high-affinity choline transporter activity by modifying its intracellular trafficking. J. Neurosci. 32, 5573–5584. 10.1523/JNEUROSCI.5235-11.2012 22514319 PMC6703479

[B9] Cuddy L. K. Seah C. Pasternak S. H. Rylett R. J. (2015). Differential regulation of the high-affinity choline transporter by wild-type and Swedish mutant amyloid precursor protein. J. Neurochem. 134, 769–782. 10.1111/jnc.13167 25970623

[B10] Cuddy L. K. Winick-Ng W. Rylett R. J. (2014). Regulation of the high-affinity choline transporter activity and trafficking by its association with cholesterol-rich lipid rafts. J. Neurochem. 128, 725–740. 10.1111/jnc.12490 24127780

[B11] DeMattos R. B. Bales K. R. Cummins D. J. Dodart J. C. Paul S. M. Holtzman D. M. (2001). Peripheral anti-A β antibody alters CNS and plasma A β clearance and decreases brain A β burden in a mouse model of Alzheimer’s disease. Proc. Natl. Acad. Sci. U S A 98, 8850–8855. 10.1073/pnas.151261398 11438712 PMC37524

[B12] Dewachter I. Filipkowski R. K. Priller C. Ris L. Neyton J. Croes S.. (2009). Deregulation of NMDA-receptor function and down-stream signaling in APP[V717I] transgenic mice. Neurobiol. Aging 30, 241–256. 10.1016/j.neurobiolaging.2007.06.011 17673336

[B13] Dodart J. C. Bales K. R. Gannon K. S. Greene S. J. DeMattos R. B. Mathis C.. (2002). Immunization reverses memory deficits without reducing brain A burden in Alzheimer’s disease model. Nat. Neurosci. 5, 452–457. 10.1038/nn842 11941374

[B14] Doody R. S. Thomas R. G. Farlow M. Iwatsubo T. Vellas B. Joffe S. (2014). Phase 3 trials of solanezumab for mild-to-moderate Alzheimer’s disease. N. Engl. J. Med. 370, 311–321. 10.1056/NEJMoa1312889 24450890

[B15] Ferguson S. M. Savchenko V. Apparsundaram S. Zwick M. Wright J. Heilman C.. (2003). Vesicular localization and activity-dependent trafficking of presynaptic choline transporters. J. Neurosci. 23, 9697–9709. 14585997 10.1523/JNEUROSCI.23-30-09697.2003 PMC6740902

[B16] Haass C. Lemere C. A. Capell A. Citron M. Seubert P. Schenk D.. (1995). The Swedish mutation causes early-onset alzheimer’s disease by β-secretase cleavage within the secretory pathway. Nat. Med. 1, 1291–1296. 10.1038/nm1295-1291 7489411

[B17] Haga T. Noda H. (1973). Choline uptake systems of rat brain synaptosomes. Biochim. Biophys. Acta 291, 564–575. 10.1016/0005-2736(73)90508-7 4690869

[B18] Hsieh H. Boehm J. Sato C. Iwatsubo T. Tomita T. Sisodia S.. (2006). AMPAR removal underlies Aβ-induced synaptic depression and dendritic spine loss. Neuron 52, 831–843. 10.1016/j.neuron.2006.10.035 17145504 PMC1850952

[B19] Hutcheon B. Brown L. A. Poulter M. O. (2000). Digital analysis of light microscope immunofluorescence: high-resolution co-localization of synaptic proteins in cultured neurons. J. Neurosci. Methods 96, 1–9. 10.1016/s0165-0270(99)00148-x 10704665

[B20] Jin M. Shepardson N. Yang T. Chen G. Walsh D. Selkoe D. J. (2011). Soluble amyloid β-protein dimers isolated from Alzheimer cortex directly induce Tau hyperphosphorylation and neuritic degeneration. Proc. Natl. Acad. Sci. U S A 108, 5819–5824. 10.1073/pnas.1017033108 21421841 PMC3078381

[B21] Kar S. Issa A. M. Seto D. Auld D. S. Collier B. Quirion R. (1998). Amyloid β-peptide inhibits high-affinity choline uptake and acetylcholine release in rat hippocampal slices. J. Neurochem. 70, 2179–2187. 10.1046/j.1471-4159.1998.70052179.x 9572306

[B22] Kristofikova Z. Ripova D. Hegnerova K. Sirova J. Homola J. (2013). Protein τ -mediated effects on rat hippocampal choline transporters CHT1 and τ-amyloid β interactions. Neurochem. Res. 38, 1949–1959. 10.1007/s11064-013-1101-5 23824558

[B23] Kristofiková Z. Tejkalova H. Klaschka J. (2001). Amyloid β peptide 1–40 and the function of rat hippocampal hemicholinium-3 sensitive choline carriers: effects of a proteolytic degradation *in vitro*. Neurochem. Res. 26, 203–212. 10.1023/A:1010908315391 11495543

[B24] Kuhar M. J. Murrin L. C. (1978). Sodium-dependent, high affinity choline uptake. J. Neurochem. 30, 15–21. 10.1111/j.1471-4159.1978.tb07029.x 340615

[B91] Legleiter J. Czilli D. L. Gitter B. DeMattos R. B. Holtzman D. M. Kowalewski T. (2004). Effect of different anti-Aβ antibodies on Aβ fibrillogenesis as assessed by atomic force microscopy. J. Mol. Biol. 335, 997–1006. 10.1016/j.jmb.2003.11.019 14698294

[B25] Li S. Hong S. Shepardson N. E. Walsh D. M. Shankar G. M. Selkoe D. (2009). Soluble oligomers of amyloid β-protein facilitate hippocampal long-term depression by disrupting neuronal glutamate uptake. Neuron 62, 788–801. 10.1016/j.neuron.2009.05.012 19555648 PMC2702854

[B26] Lorenzen A. Samosh J. Vandewark K. Anborgh P. H. Seah C. Magalhaes A. C.. (2010). Rapid and direct transport of cell surface APP to the lysosome defines a novel selective pathway. Mol. Brain 3:11. 10.1186/1756-6606-3-11 20409323 PMC2868040

[B27] Miles L. A. Crespi G. A. Doughty L. Parker M. W. (2013). Bapineuzumab captures the N-terminus of the Alzheimer’s disease amyloid-β peptide in a helical conformation. Sci. Rep. 3:1302. 10.1038/srep01302 23416764 PMC3575012

[B28] Nitsch R. M. Slack B. E. Wurtman R. J. Growdon J. H. (1992). Release of Alzheimer amyloid precursor derivatives stimulated by activation of muscarinic acetylcholine receptors. Science 258, 304–307. 10.1126/science.1411529 1411529

[B29] Okuda T. Haga T. (2000). Functional characterization of the human high-affinity choline transporter. FEBS Lett. 484, 92–97. 10.1016/s0014-5793(00)02134-7 11068039

[B30] Panza F. Frisardi V. Imbimbo B. P. Seripa D. Paris F. Santamato A.. (2011). Anti-β amyloid immunotherapy for Alzheimer’s disease: focus on bapineuzumab. Curr. Alzheimer Res. 8, 808–817. 10.2174/156720511798192718 21592055

[B31] Parikh V. Bernard C. S. Naughton S. X. Yegla B. (2014). Interactions between Aβ oligomers and presynaptic cholinergic signaling: age-dependent effects on attentional capacities. Behav. Brain Res. 274, 30–42. 10.1016/j.bbr.2014.07.046 25101540 PMC4179990

[B32] Pedersen W. A. Kloczewiak M. A. Blusztajn J. K. (1996). Amyloid β-protein reduces acetylcholine synthesis in a cell line derived from cholinergic neurons of the basal forebrain. Proc. Natl. Acad. Sci. U S A 93, 8068–8071. 10.1073/pnas.93.15.8068 8755604 PMC38876

[B33] Pinthong M. Black S. A. Ribeiro F. M. Pholpramool C. Ferguson S. S. Rylett R. J. (2008). Activity and subcellular trafficking of the sodium-coupled choline transporter CHT is regulated acutely by peroxynitrite. Mol. Pharmacol. 73, 801–812. 10.1124/mol.107.040881 17971421

[B35] Pul R. Dodel R. Stangel M. (2011). Antibody-based therapy in Alzheimer’s disease. Expert Opin. Biol. Ther. 11, 343–357. 10.1517/14712598.2011.552884 21261567

[B36] Reed M. N. Hofmeister J. J. Jungbauer L. Welzel A. T. Yu C. Sherman M. A.. (2012). Cognitive effects of cell-derived and synthetically-derived Aβ oligomers. Neurobiol. Aging 32, 1784–1794. 10.1016/j.neurobiolaging.2009.11.007 20031278 PMC2895944

[B37] Ribeiro F. M. Black S. A. G. Cregan S. P. Prado V. F. Prado M. A. M. Rylett R. J.. (2005). Constitutive high-affinity choline transporter endocytosis is determined by a carboxyl-terminal tail dileucine motif. J. Neurochem. 94, 86–96. 10.1111/j.1471-4159.2005.03171.x 15953352

[B38] Rylett R. J. Schmidt B. M. (1993). Regulation of the synthesis of acetylcholine. Prog. Brain Res. 98, 161–166. 8248504 10.1016/s0079-6123(08)62394-8

[B39] Salloway S. Sperling R. Fox N. C. Blennow K. Klunk W. Raskind M.. (2014). Two phase 3 trials of Bapineuzumab in mild-to-moderate Alzheimer’s disease. N. Engl. J. Med. 370, 322–333. 10.1056/NEJMoa1304839 24450891 PMC4159618

[B40] Sarter M. Parikh V. (2005). Choline transporters, cholinergic transmission and cognition. Nat. Rev. Neurosci. 6, 48–56. 10.1038/nrn1588 15611726

[B41] Schenk D. (2002). Amyloid-β immunotherapy for Alzheimer’s disease: the end of the beginning. Nat. Rev. Neurosci. 3, 824–828. 10.1038/nrn938 12360327

[B42] Schneeberger A. Mandler M. Otawa O. Zauner W. Mattner F. Schmidt W. (2009). Development of AFFITOPE vaccines for Alzheimer’s disease (AD)—from concept to clinical testing. J. Nutr. Health Aging. 13, 264–267. 10.1007/s12603-009-0070-5 19262965 PMC12876384

[B43] Schroeter S. Khan K. Barbour R. Doan M. Chen M. Guido T.. (2008). Immunotherapy reduces vascular amyloid-β in PDAPP mice. J. Neurosci. 28, 6787–6793. 10.1523/JNEUROSCI.2377-07.2008 18596154 PMC6670967

[B44] Shankar G. M. Li S. Mehta T. H. Garcia-Munoz A. Shepardson N. E. Smith I.. (2008). Amyloid-β protein dimers isolated directly from Alzheimer’s brains impair synaptic plasticity and memory. Nature Med. 14, 837–842. 10.3410/f.1115634.578903 18568035 PMC2772133

[B45] Spencer B. Masliah E. (2014). Immunotherapy for Alzheimer’s disease: past, present and future. Front. Aging Neurosci. 6:114. 10.3389/fnagi.2014.00114 24959143 PMC4051211

[B90] Tayeb H. O. Murray E. D. Price B. H. Tarazi F. I. (2013). Bapineuzumab and solanezumab for Alzheimer’s disease: is the ‘amyloid cascade hypothesis’ still alive? Expert Opin. Biol. Ther. 13, 1075–1084. 10.1517/14712598.2013.789856 23574434

[B46] Thinakaran G. Teplow D. B. Siman R. Greenberg B. Sisodia S. S. (1996). Metabolism of the “Swedish” amyloid precursor protein variant in neuro2a (N2a) cells. Evidence that cleavage at the “β-secretase” site occurs in the golgi apparatus. J. Biol. Chem. 271, 9390–9397. 10.1074/jbc.271.16.9390 8621605

[B47] Townsend G. M. Shankar G. M. Mehta T. Walsh D. M. Selkoe D. J. (2006). Effects of secreted oligomers of amyloid β-protein on hippocampal synaptic plasticity: a potent role for trimers. J. Physiol. 572, 477–492. 10.1113/jphysiol.2005.103754 16469784 PMC1779683

[B48] Walsh D. M. Klyubin I. Fadeeva J. V. Cullen W. K. Anwyl R. Wolfe M. S.. (2002). Naturally secreted oligomers of amyloid β protein potently inhibit hippocampal long-term potentiation *in vivo*. Nature 416, 535–539. 10.1038/416535a 11932745

[B49] Wang B. Yang L. Wang Z. Zheng H. (2007). Amyloid precursor protein mediates presynaptic localization and activity of the high-affinity choline transporter. Proc. Natl. Acad. Sci. U S A 104, 14140–14145. 10.1073/pnas.0704070104 17709753 PMC1955810

[B50] Young-Pearse T. L. Bai J. Chang R. Zheng J. B. LoTurco J. J. Selkoe D. J. (2007). A critical function for β-amyloid precursor protein in neuronal migration revealed by *in utero* RNA interference. J. Neurosci. 27, 14459–14469. 10.1523/JNEUROSCI.4701-07.2007 18160654 PMC6673432

[B51] Zago W. Buttini M. Comery T. A. Nishioka C. Gardai S. J. Seubert P.. (2012). Neutralization of soluble, synaptotoxic amyloid β species by antibodies is epitope specific. J. Neurosci. 32, 2696–2702. 10.1523/jneurosci.1676-11.2012 22357853 PMC6621896

